# Barriers and facilitators to opioid agonist treatment (OAT) engagement among individuals released from federal incarceration into the community in Ontario, Canada

**DOI:** 10.1080/17482631.2022.2094111

**Published:** 2022-07-05

**Authors:** Cayley Russell, Michelle Pang, Frishta Nafeh, Shanna Farrell Macdonald, Dena Derkzen, Jürgen Rehm, Benedikt Fischer

**Affiliations:** a Institute for Mental Health Policy Research, Centre for Addiction and Mental Health (CAMH), Toronto, Ontario, Canada; b Policy Sector, Research Branch, Correctional Service of Canada, Ottawa, Ontario, Canada; c Department of Psychiatry, University of Toronto, Toronto, Ontario, Canada; d Dalla Lana School of Public Health, University of Toronto, Toronto, Ontario, Canada; e Campbell Family Mental Health Research Institute, Centre for Addiction and Mental Health, Toronto, Ontario, Canada; f Institute of Medical Science (IMS), University of Toronto, Toronto, Ontario, Canada; g Institut für Klinische Psychologie und Psychotherapie, Technische Universität Dresden, Dresden, Germany; h Department of International Health Projects, Institute for Leadership and Health Management, I.M. Sechenov First Moscow State Medical University, Moscow, Russia; i Centre for Applied Research in Mental Health and Addiction (CARMHA), Faculty of Health Sciences, Simon Fraser University, Vancouver, British Columbia, Canada; j Faculty of Medical and Health Sciences, University of Auckland, Auckland, New Zealand; k Department of Psychiatry, Federal University of São Paulo (UNIFESP), São Paulo, Brazil

**Keywords:** Addiction, Canada, community release, corrections, opioid agonist treatment, opioid use disorder, parole, prison, re-integration, re-incarceration, substance use

## Abstract

**Introduction:**

Correctional populations with opioid use disorder experience increased health risks during community transition periods. Opioid Agonist Treatment (OAT) can reduce these risks, but retention is a key challenge. This study addresses a knowledge gap by describing facilitators and barriers to OAT engagement among federal correctional populations released into the community in Ontario, Canada.

**Methods:**

This article describes results from a longitudinal mixed-methods study examining OAT transition experiences among thirty-five individuals released from federal incarceration in Ontario, Canada. Assessments were completed within one year of participants’ release. Data were thematically analyzed.

**Results:**

The majority (77%) of participants remained engaged in OAT, however, 69% had their release suspended and 49% returned to custody. Key facilitators for OAT engagement included flexibility, positive staff rapport, and structure. Fragmented OAT transitions, financial OAT coverage, balancing reintegration requirements, logistical challenges, and inaccessibility of ‘take-home’ OAT medications were common barriers.

**Conclusions:**

Post-incarceration transition periods are critical for OAT retention, yet individuals in Ontario experience barriers to OAT engagement that contribute to treatment disruptions and related risks such as relapse and/or re-incarceration. Additional measures to support community OAT transitions are required, including improved discharge planning, amendments to OAT and financial coverage policies, and an expansion of OAT options.

## Introduction

Correctional populations globally, and in Canada specifically, experience an excess burden of health issues, such as elevated prevalence of chronic physical and mental health challenges. These include problematic drug use, namely substance use disorders and specifically opioid use disorder (OUD; Beaudette, 2013; Fazel et al., 2016, 2017; Kouyoumdjian et al., 2014; Kouyoumdjian, Schuler et al., 2016; Mullins & Farrell, 2012). For instance, nearly four-out-of-five individuals admitted to federal correctional institutions in Canada between 2010–2014 reported a substance use issue, with 14% of men and 25% of women indicating past-year opioid use, as well as high rates of polysubstance and injection drug use (Kelly & Farrell Macdonald, 2015a, 2015b). These data are also likely an underrepresentation in the context of the current opioid epidemic in Canada (Caulkins et al., 2021; Special Advisory Committee on the Epidemic of Opioid Overdoses, 2020), particularly since involvement in the criminal justice system has been found to increase with intensity and frequency of opioid use (Winkelman et al., 2018).

Many of the health and substance use challenges experienced by correctional populations are exacerbated upon release from prison into the community (Hall & Farrell, 2018; Hopkin et al., 2018). For instance, administrative data from Ontario demonstrate surges in emergency department usage and hospitalization among individuals upon community release (Kouyoumdjian, Cheng et al., 2018). Moreover, correctional populations have a significantly higher risk of premature mortality post-incarceration (Binswanger et al., 2007), commonly due to drug poisonings (including opioid overdose) resulting from decreased tolerance to substances while incarcerated (Fox, Moore et al., 2019; Maradiaga et al., 2016). In Canada, data confirm elevated rates of (fatal and non-fatal) opioid overdoses post-incarceration (Groot et al., 2016; Kouyoumdjian, Kiefer et al., 2016; Madadi et al., 2013). Additional literature exists observing the chronic relapsing nature of OUD, related risk factors (e.g., injection drug use), and the high incidence of substance use relapse, recidivism, non-fatal overdoses, and treatment disruptions among correctional populations following release from incarceration (Brinkley-Rubinstein et al., 2017; Brinkley-Rubinstein, Cloud et al., 2018; Brinkley-Rubinstein, Zaller et al., 2018; Chamberlain et al., 2019; Keen et al., 2020; Kirwan et al., 2019; Martin et al., 2019; Murphy et al., 2018; Western & Simes, 2019; Winter et al., 2016). Therefore, taken together, the extant literature provides strong evidence that release from imprisonment represents a critical period for incarcerated individuals’ health, specifically for those with OUD.

OUD is a complex disorder that is predominantly managed via pharmacological maintenance with opioid agonist treatment (OAT) (typically methadone and/or buprenorphine-naloxone [Suboxone] formulations in Canada) to prevent withdrawal and reduce risky opioid use (Bruneau et al., 2018; Stotts et al., 2009; Strang et al., 2020). OAT is an evidence-based and safe treatment that has been associated with beneficial outcomes among correctional populations upon community release. These include lowered illicit opioid use, overdoses, mortality, recidivism and re-incarceration, as well as increased health care and addiction treatment engagement, and better social re-integration (Akinsemolu et al., 2011; Cropsey et al., 2005; Crowley & Van Hout, 2017; Glanville et al., 2021; Hedrich et al., 2012; Malta et al., 2019; Moore et al., 2019; Perry et al., 2015; Schwartz et al., 2018; Sharma et al., 2016; Stallwitz & Stover, 2007). Confirmatory data on these benefits for Canadian-specific correctional populations also exist (Farrell-Macdonald et al., 2014; MacSwain et al., 2013; Russolillo, Moniruzzaman, McCandless et al., 2018; Russolillo, Moniruzzaman, Somers et al., 2018). The initiation of OAT within Canadian federal correctional institutions, combined with continuous linkage to OAT treatment upon release, is therefore essential to support the health and well-being of this underserved population with complex needs (Binswanger et al., 2011; Larney & Dolan, 2009; McKenzie et al., 2009; Murphy & Sapers, 2020; Nunn et al., 2009).

OAT has been available in Canadian federal correctional institutions for over two decades (Correctional Service Canada, 2019, 2021), however uptake and retention upon release remain limited (Bozinoff et al., 2018). In the context of the ongoing national opioid crisis, recent efforts have occurred to expand OAT initiation among Canadian federally incarcerated individuals and institutions now offer methadone, buprenorphine/naloxone, and, as of 2019, extended-release injectable buprenorphine (Correctional Service Canada, 2019, 2021, 2020). The majority of federally incarcerated individuals in Canada are eventually released into the community, usually under a conditional release (e.g., day/full parole, statutory release [after serving 2/3 of sentence], long-term supervision orders, etc.). During this time they are required to abide by specific release conditions (e.g., refrain from substance use, reside within a community-based residential facility [i.e., a “halfway house”], etc.), with the ultimate goal of rehabilitation and reintegration into society (Correctional Service Canada, 2007; Macmadu & Rich, 2015; McLeod & Martin, 2018). Continual engagement in OAT throughout this supervised community re-entry period is crucial, however, a myriad of factors may influence OAT commitment and retention. For instance, system- and structural-level factors have been identified to uniquely impact and hinder OAT retention. These include sub-par release planning, administrative issues, challenges securing housing and employment, negative interactions with the correctional system and/or parole officers, and a lack of transportation (Bunting et al., 2018; Hu et al., 2020; Jamin et al., 2021; Joudrey et al., 2019; Vail et al., 2021; Velasquez et al., 2019). Individual-level (i.e., psychological or social/behavioural) barriers have also been identified, including motivation, mental and physical health conditions, stigma, stress, and poor social support including negative influences of substance-using peers which commonly contribute to substance use relapse (Binswanger et al., 2012; Jamin et al., 2021; Owens et al., 2018). Qualitative data confirm that exposure to drugs, when combined with overwhelming reintegration requirements, often contribute to opioid use relapse and influence OAT treatment decisions (Fox et al., 2015).

While basic descriptive data regarding OUD and OAT engagement among correctional populations in Canada are available (Bozinoff et al., 2018; Farrell-Macdonald et al., 2014; MacSwain et al., 2013), little information exists related to the release experiences of this population. Understanding the specific circumstances and nuances that influence OAT treatment adherence, substance use relapse, and broader social reintegration, including any specific barriers and/or facilitators to continuous OAT engagement is therefore important and required. Not only is this information necessary to develop potential public health interventions and measures to help support this high-risk population, but it is also integral to being able to mitigate adverse post-release morbidity and mortality (Gisev et al., 2019). In order to address this critical knowledge gap, this article describes findings from a longitudinal, mixed-methods study that examined OAT transition experiences among a regional sample of federally incarcerated individuals with OUD following release into the community in Ontario, Canada.

## Methods

### Recruitment and sample

This article focuses on the follow-up results of a longitudinal observational mixed-methods cohort study examining community OAT transitions and engagement among a sample of federally incarcerated individuals with OUD released into the community within the following year in Ontario, Canada. Participants were initially recruited from the seven federal Correctional Service Canada (CSC) institutions located in Ontario (six housing male inmates, one housing female inmates). An initial baseline assessment was conducted with 46 participants who met our inclusion criteria between January to March, 2019 (see, Russell et al., 2021 for full baseline methods and results). For baseline participant recruitment, CSC’s Research Branch provided a list of potential participants who met study eligibility criteria (i.e., individuals engaged in OAT for at least three months, with pending community release dates within the next six months) to site contacts at each insitution. Site contacts then shared the study flyer/details with these participants and posted it in the general healthcare area for anyone who was interested to call the toll-free study line. Interested individuals then presented for eligibility screening during the first day of data collection at each institution. Participants provided their institutional fingerprint serial (FPS) number and a pseudonym, and were given a study ID number to identify them on all study documents. Participants who met eligibility and were interested in participating were enrolled in the study and scheduled to complete the assessment later that day/week. At initial baseline assessment, participants underwent an informed consent procedure where the research team obtained explicit consent to use participants’ FPS numbers to link additional CSC administrative data with survey data, as well as to contact their individual parole officers upon their release to arrange the follow-up assessment.

Contact with prospective follow-up study participants was facilitated through two main approaches: 1) institutional CSC staff and parole officers providing contact information; and/or 2) research staff directly contacting participants who had provided their contact information during the baseline assessment. Research staff contacted prospective research participants via telephone or email to confirm study interest/participation and to arrange a time and location to conduct the in-person follow-up assessment. Baseline participants who had reached warrant expiry (i.e., completed their community release period prior to the follow-up assessment) were contacted for the follow-up assessment using contact information provided; however, participants were considered lost to follow-up after several unsuccessful contact attempts were made.

A total of n = 35 participants completed the follow-up assessment, representing a 76% participant retention rate from the original baseline sample (n = 46). Among those lost to follow-up, seven individuals had completed their sentence and could not be contacted, and an additional four had not been released during the study period. The average time from release to follow-up assessment completion was five months; however, the timespan ranged from one to ten months.

### Follow-up assessments

The follow-up assessment consisted of a brief (15–30 minute) quantitative survey followed by a (30–60 minute) qualitative semi-structured, audio-recorded, one-on-one interview consisting of six open-ended questions and relevant probes focusing on post-release OAT experiences and engagement, including barriers and facilitators (see Appendix A for interview guide). Notably, all self-report data collected focused on the “past 30 days” and thus provided a temporal snapshot of the participants’ post-release period.

Complementary administrative data was obtained from a CSC database (i.e., the Offender Management System [OMS]) using participants’ individual FPS numbers. The OMS maintains individual records and socio-demographic data of all incarcerated individuals during incarceration and post-release while under community supervision (Correctional Service Canada, 2013).

All follow-up assessments were conducted between 1 October 2019 and March 16^th^, 2020, in field locations across Ontario, and occurred within the first year of each participant’s community release. Interviews were conducted by a member of the research team trained in qualitative interviewing (CR). Locations for study assessment meetings were set based on participant convenience in addition to safety and privacy/confidentiality considerations. In-person assessments took place in private rooms at individual parole offices (n = 7), halfway houses (n = 9), residential/inpatient substance use treatment facilities (n = 2), or in correctional institutions (n = 6) for those who had returned to federal custody following community release. Telephone assessments were conducted for participants who had either moved out-of-province or where an in-person meeting was not feasible (n = 11). Upon follow-up assessment completion, participants were provided with a $50 Visa gift card honoraria for their time and expertise.

### Data processing and analysis

All personal and identifying information were removed from data collected. The only identifying information obtained from participants was their FPS number and a pseudonym, and all participants were assigned an anonymous study code for data management and identification purposes. Quantitative survey data were entered into an encrypted Excel database for cleaning and analysis, which included basic descriptive counts and frequencies. Interview audio recordings were transcribed verbatim and imported into qualitative data management software (NVivo 12). Basic descriptive statistics (e.g., mean and frequency counts) were analysed for participant characteristics based on both the survey and CSC administrative data.

All qualitative interview data underwent an inductive thematic analysis process where initial themes were developed based on the study’s research questions, and a preliminary codebook was developed in Excel (Creswell & Creswell, 2017). One member of the research team open-coded the transcripts based on the initial codebook to identify common responses to our research questions (specifically facilitators and barriers to OAT and community reintegration). Following extensive discussion among members of the research team, the initial codes were then refined and further categorized and applied to the data. Data were then coded line-by-line to ensure all themes were being accurately captured, and any additional codes were subsequently added to the codebook as part of the iterative coding process (Williams & Moser, 2019). In order to ensure transparency and accuracy in data analysis, the research team relied on inter-coder reliability whereby an independent coder coded a sub-sample of the transcripts (O’Connor & Joffe, 2020), and any codebook revisions and coding queries were resolved with the team based on ongoing discussion. The final qualitative themes and sub-themes presented were informed by multiple participants conveying similar sentiments and statements until data saturation was met (Fusch & Ness, 2015; Saunders et al., 2018). All themes are narratively summarized and are further illustrated and substantiated by select participant quotes.

### Ethics

This study was approved by The Centre for Addiction and Mental Health (CAMH) institutional review board (REB# 013-2018). All participants provided explicit written informed consent during the baseline assessment to participate in the follow-up assessment, and for anonymous/aggregate administrative CSC data linkage.

## Results

### Quantitative results

The majority of participants identified as white (69%), men (83%), with a mean age of 36 years. table 1 outlines release, drug use, and OAT information. All participants had release conditions that included abstaining from substance use and avoiding certain “risk groups” (e.g., criminal associates, victims). For four-fifths, participation in a treatment program/counselling was a requirement. In regards to substance use, among those who had submitted urinalysis tests while in the community (91%), more than three-fifths (63%) tested positive for an illicit substance, with opioids as the most commonly used substance. Three-quarters (77%) of the sample remained engaged in OAT after release, with most (59%) on buprenorphine-naloxone-based OAT. Two-thirds (69%) of participants had their release suspended, while half (49%) returned to custody; violating substance use-related conditions were cited as the reason in 88% and 82% of these cases, respectively.

**Table 1.** Study sample release, drug use, and OAT information (n=35)

**Characteristics**	**% (n)**
**Release conditions** Abstain from substance use Avoid certain people (criminal associates, victims) Participate in treatment program/counselling Residency	100 (35) 100 (35) 80 (28) 34 (12)
**Community urinalysis** Positive for illicit substances+ Opioids	91 (32) 63 (20 of 32) 50 (10 of 20)
**Medical marijuana prescription**^α^	23 (8)
**OAT engagement**^α^ Methadone Buprenorphine-naloxone	77 (27) 40 (11 of 27) 59 (16 of 27)
**Suspension of release** Substance use as a factor in suspension	69 (24) 88 (21 of 24)
**Return to custody** Substance use as a factor in return to custody	49 (17) 82 (14 of 17)

^+Indicator is not mutually-exclusive; α Data derived from quantitative survey, and only indicative of past 30 days at time of assessment. The 3 participants without community urinalysis all self-reported illicit substance (with 2 including opioid) use. Of the 10 participants who tested positive for illicit substance (excluding opioid) use, 6 were OAT-engaged; of the 10 participants who tested positive for opioid use, 7 were OAT-engaged. Release suspensions are temporary interruption of release, typically for a breach of conditions; ‘return to custody’ is a revoked release. If an individual does not meet their community requirements or re-offends, their release can be suspended (i.e., a temporary interruption of their release, typically for a breach of conditions). The Parole Board of Canada (PBC) must then decide whether to cancel the suspension (i.e., return the individual to the community based on the circumstances of the suspension), or revoke the release (i.e., the individual returns to federal custody; for individuals on discretionary release [day or full parole], they will then have to re-apply for release; for those on statutory release, CSC reviews and recalculates when they will be re-released.^

### Qualitative results

Participants provided both positive and negative perspectives regarding their community reintegration experiences, and elaborated on various factors that had impacted their release and served as either barriers and/or facilitators to connecting and engaging with OAT. These qualitative data are presented under the following major and respective sub-headings: facilitators for post-release OAT engagement, including the perceived benefits of OAT, flexibility of OAT provision and staff rapport, and OAT program structure; and barriers to post-release OAT engagement, including fragmented community OAT transitions, financial OAT coverage, balancing OAT and other reintegration requirements, logistical challenges and unfavourable OAT clinic dynamics, and access to take-home OAT medications.

### Facilitators for post-release OAT engagement

#### Perceived benefits of OAT

Most participants who remained engaged in OAT following community release described both physiological and psychological benefits of OAT engagement. Many participants described initially becoming addicted to opioids through legitimate opioid prescriptions for physical injuries. As such, the capability of OAT to alleviate physical pain in addition to other issues such as sleep or emotional problems, while also reducing opioid withdrawal symptoms, was perceived as beneficial:

*“Methadone has helped a lot. Like since being on methadone, I’ve been sleeping almost every night … sleeping regularly has helped a lot with my mental health. My anxiety levels are down. I’m able to control my anger and cope with all my emotions better. Also, I’ve been eating regularly which I never really did before. It’s helped me with my back pain. Definitely helps with cravings and triggers and stuff.” (Participant 36*^1^)

Specifically, some participants explained that OAT worked as a psychological disincentive for illicit opioid use. This was a common theme for those on buprenorphine-naloxone, many of whom believed that its “abuse-deterrent” formulation (i.e., the naloxone component) would precipitate withdrawal if they used or injected opioids. As such, many participants preferred buprenorphine-naloxone to methadone, even among those who still frequently experienced opioid-related cravings and triggers:

*“I get cravings every day. Every single day. The cravings are always there, even with the Suboxone. But the way I look at it is like, my downfall was opiates. With the Suboxone, I keep it in my head that if I do any opiates, I’ll go into withdrawal. So that keeps me away from the opiates.” (Participant 12)*

Many participants perceived OAT as a key factor for refraining from illicit opioid use, desisting from crime, and ultimately achieving their personal goals for community life. Even among participants with complex opioid use histories, many indicated that OAT engagement had been integral for disrupting their substance use habits and patterns:

*“I was in the penitentiary 10 years ago, I was in and out of provincial jail, and I would go back to using opiates. Upon my release … I was always right back into the cycle of using opiates, constantly. And from getting on the Suboxone program [while incarcerated], I think it was the best thing I ever did. I should have did it [sic] years ago. It has changed me, period.” (Participant 01)*

#### Flexibility of OAT provision and staff rapport

While participants described the benefits of OAT towards reaching community reintegration goals and desisting from substance use, many elaborated on specific favourable aspects of community-based OAT care provision. For example, many described the flexibility to choose the type of OAT formulation that worked best for them, and working with OAT providers to jointly set comfortable dosage levels as desirable factors which contributed to improved OAT adherence. Participants further explained the importance of developing positive relationships and rapport with OAT program staff (e.g., physicians, nurses):

*“I really feel supported by my methadone clinic. They’re really working with me to make sure that it’s the right dose, and that I’m there. It’s just a really good clinic, they know you by face. The doctor is so cool. They’re all amazing, I have no problem with them at all.” (Participant 32)*

Several participants underscored the important role of OAT and/or related addiction program/clinic staff members who had provided positive OAT care experiences and thus supported their engagement and continued adherence to OAT in the community. Others elaborated on the significance of trusted relationships with OAT providers. Participants expressed appreciation for OAT providers who they perceived as genuinely supportive:

*“The doctor I have right now is great because they understand and they didn’t try and get me in trouble when I had a [positive urinalysis] … They’re there to help me, not send me back to jail. And that’s a big thing. Jail doesn’t help you. This stuff helps you, right?” (Participant 13)*

#### Structure of OAT programs

Some participants expressed that the rigorous structure of the OAT program helped facilitate treatment adherence. Generally, participants were required to visit OAT clinics/pharmacies daily to obtain their OAT medications and/or provide urinalysis samples for monitoring substance use. While some participants described mixed feelings towards stringent clinic requirements and perceived the frequent attendance as burdensome, others recognized that these requirements helped them remain committed and accountable. In particular, the requirement to provide a negative’ (i.e., drug-free) urinalysis test was described as a crucial measure for both OAT adherence and desistance from substance use, as detailed by a participant who had relapsed during their community release:

*“I didn’t [use drugs while I was on Suboxone] or piss dirty once. I did drink while I was on it. Like I would drink on the weekends, right? But, I didn’t use any drugs or anything. Just the amount of pain-in-the-ass it was to have to go down every week to provide the urine sample, which is fine. I didn’t feel I needed it anymore, you know? And it turns out that I couldn’t have been more wrong, because it provided motivation to stay clean.” (Participant 06)*

However, while participants described positive experiences with OAT engagement upon release, many also described barriers to OAT engagement and related adverse consequences.

### Barriers to post-release OAT engagement

#### Fragmented community OAT transitions

The most common barrier to post-release OAT engagement reported by participants was fragmented community OAT care transitions. Nearly a quarter of participants experienced issues connecting to their designated community-based OAT providers upon release. In several instances, crucial information or paperwork (e.g., proof of identification or last dose) was not conveyed or was lost during the community transfer. Consequently, some participants presented to OAT clinics and were denied treatment, thus resulting in treatment interruptions. Some participants had to wait several days before they could obtain their medication, while others were able to receive it later that first day, but only after undertaking onerous administrative processes such as contacting their parole officers and/or various OAT clinics to follow-up:

*“[The prescription] never got faxed over or something, it didn’t work out. So [the OAT clinic] closes at five o’clock. So because I didn’t get my dose, they had to call [a different pharmacy] and get everything set up there through CSC, and it was like 11 o’clock at night before I got it. They didn’t have proof of last dose, so that’s what didn’t get shipped or faxed or whatever. So, it was a big hassle because at that time, my curfew [at the halfway house] was nine o’clock. So, on my first day I had to get like a curfew extension, which had to be approved through the police, and it was hell.” (Participant 15)*

Some participants explained that they did not possess the valid forms of identification (e.g., health cards) required to access OAT in the community, resulting in clinics delaying or denying their treatment. In light of these and related issues, a number of participants suggested that they should be provided with an OAT prescription upon community release as this would reduce obstacles when presenting at community OAT clinics to obtain their medication:

*“I guess just getting more set up for the release. Like having the doctor from the jail give you a prescription for the first three or four days so that you’re guaranteed to have your [OAT] medication when you’re getting out. If they let you leave with a prescription, maybe that would be a little bit easier.” (Participant 12)*

While many participants identified fragmented connections to OAT care providers as problematic and frustrating, for others, these factors were the main driving force behind the decision to discontinue OAT.

#### Financial OAT coverage

Another substantial barrier to OAT engagement involved financial challenges, specifically in regards to the costs of OAT care and medications. In Ontario, OAT medication costs post-release are typically covered by correctional institutions for a limited period (usually until alternative coverage can be arranged), after which individuals must pay out-of-pocket or seek support through government/social assistance programs (e.g., welfare, disability, low-income prescription support) or employer-sponsored benefit programs. However, many participants did not qualify for employer benefits, and participants described encountering major issues applying for and receiving financial support (e.g., including being ineligible due to requirements for valid government identification and/or not having completed federal income tax returns). Taken together, these issues left many participants without adequate financial coverage for OAT, which placed them in the vulnerable position of having to pay extensive amounts out-of-pocket (typically between $5.00—$20.00 CAD per dose per day):

*“When I was released, the halfway house covers [OAT] for three months, but then you got to apply for the Trillium benefit thing. But what happened was on the three-month mark I was supposed to send an application to get it so I could be covered … but instead of putting the correct date to the next three months, I put when I got released … they didn’t explain it to me properly. So, after weeks of that, I started paying out of my own pocket. But thank god I was working because it was pretty much $9 a dose every day. I paid almost $400 for the month of September.” (Participant 18)*

Some participants resorted to borrowing money from friends or family. Others shared that they had contemplated committing crimes to generate money to pay for OAT medications, thus risking the possibility of re-incarceration. For many participants, economic barriers such as gaps in financial coverage for OAT posed a direct and significant barrier to OAT engagement:

*“I’m trying to fill out the papers for myself because I am eligible for [government disability assistance]. Right now, I’m just running up debts, and it’s not good. Just to get my [OAT] medication for the first two weeks, I was spending $60 a day … I didn’t even want to go get my medication half the time because I’m like, I have to pay $20 there [and] $20 back for the Uber, and then $20 for the medication. It’s like, $60 every single day.” (Participant 12)*

#### Balancing OAT and other reintegration requirements

Many participants described how the burden of OAT adherence (i.e., visiting the OAT clinic daily, providing weekly urinalyses, repeat prescription renewals, etc.) was exacerbated by their other reintegration requirements (e.g., curfews, obtaining/retaining employment and housing, weekly/monthly parole meetings, substance use treatment and/or correctional programing, counselling, etc.,). This was particularly the case for those who were employed; some participants were luckily able to alter their work schedules (e.g., switch to night shifts or request time off work) to accommodate daily OAT adherence, however, others described substantial challenges doing so, including experiencing subsequent interpersonal conflicts and/or job loss. For instance, one participant described challenges associated with trying to adhere to their OAT while also maintaining full-time employment:

*“[The OAT doctor] is only in the clinic on Thursdays, and they’re closed at 5pm. And you have to make in that window. And, like I said to him, when I’m in a union job, we work every day, five in the morning till six, seven at night. There is no way possible that I could book off an afternoon every Thursday or every other Thursday to do a urinalysis and pick up a [OAT prescription]. It doesn’t work with people who are working, and it shouldn’t be like that because you’re clean, you’re moving ahead, you’re moving forward with your life.” (Participant 01)*

Moreover, some participants expressed that they did not feel comfortable disclosing their OAT status with their employer, creating additional OAT-related barriers:

*“I’ve got to figure out how to get this Suboxone out of my way, so I can get it at night. I’ll have to talk to my boss about it. I find it interrupting [sic] for me to go there first thing in the morning, you know? I mean the fact that I got to get up at six o’clock in the morning, every single day. [My work] don’t know about it … there’s lots of stuff like that I like to keep private.” (Participant 14)*

For some, difficulties balancing release requirements with OAT adherence was a catalyst for treatment disruptions or ceasing OAT.

#### Logistical challenges and unfavourable OAT clinic dynamics

In addition to difficulties balancing OAT and other reintegration requirements, many participants described an array of logistical challenges that impeded OAT engagement. Some participants lived or worked far distances from the nearest OAT clinic and subsequently described related issues such as a lack of reliable and/or affordable transportation to get there, especially during unfavourable weather conditions. For those who resided in rural or non-urban areas, OAT clinics were rare and had limited hours of operation (e.g., closed early and/or on weekends) which posed significant challenges to accessibility. As such, some participants indicated the need for more clinics to be available and for extended operational hours:

*“I just think that you should be able to have afterhours where people working can go in and do their urinalysis. Either have a clinic that is open late, even if it was one day a week … ‘till say 8:00pm. So, where people working day jobs could still make it there, do their urine test, show that they’re clean, get their script, and then move on with the month.” (Participant 01)*

For some, these logistical challenges resulted in the discontinuation of OAT altogether:

*“[Getting OAT] was a real big hassle. I would have to get dropped off at the [pharmacy after work] … and then get my [OAT medication], and then I’d have to walk almost an hour and a half back to the halfway house, or wait for the bus, which basically took the same amount of time. I’m just done with it. I didn’t want to do it no more. I didn’t want to walk every day after work for an hour, it was just way too much.” (Participant 07)*

Other unfavourable OAT clinic dynamics included easy access to illegal drugs. Specifically, OAT locations were often described as “hot spots” for drug activity and participants were wary of running into peers and/or individuals who were actively using or dealing drugs in the immediate vicinity. Some participants expressed reluctance towards attending their OAT appointments for this reason; for some, this was the primary motivation to wean off and/or cease OAT prior to community release. Others explained that these circumstances created “triggers” which caused them to relapse into substance use. For instance, one participant described being persuaded into using drugs following an encounter with an acquaintance near their OAT clinic:

*“I was a little bit worried about coming back to [city name]. I have been using and selling drugs here for the past 10 years. So, I was just worried about running into people. So I went to the methadone clinic and I ran into someone that I used to sell drugs to, and they were smoking crack in the back of the clinic there, and offered me some. I resisted at first … and they offered again and I took it.” (Participant 30)*

#### Access to “take-home” OAT medications

Lastly, participants described that many of the previously identified barriers to OAT engagement were compounded by institutional and administrative policies enforced at some halfway houses which prohibited possession of take-home OAT medication doses (i.e., “carries”) while residing on the premises. Thus, these participants—many of whom had maintained their OAT regimens and abstained from substance use during this time—were still required to attend their daily OAT appointments in order to receive their medication. Participants described contempt for these “no-carry” policies and considered them arbitrary, especially since many halfway houses would permit storage of other types of medications, including other opioids and/or medical marijuana:

*“So here at the [halfway] house, they don’t allow Suboxone or methadone. So it’s kind of provided me with a roadblock for the reason that I have to go to the clinic every day, which doesn’t open until [late] on the weekend. So it kind of affects the ability for me to have a day job. I’m not saying it’s prevented me from having a job period, it just makes it more difficult. Yeah, they don’t allow carries here. But they allow prescription marijuana. Even my worker here, he says that they keep hydromorphs [prescribed opioids] here for people.” (Participant 08)*

Others described additional challenges related to obtaining OAT carries, for instance, the time and effort required to “work up” to a full week (or longer) supply of carries, or not being aware that some OAT clinics required substantial advance notice to issue carries. For one participant, the inability to receive carries for a weekend trip resulted in withdrawal symptoms, and ultimately, relapse into substance use:

*“Well I got a weekend pass for four days, and usually I get carries to go home with, and they never gave me any carries. So I ended up using [drugs] because I didn’t have my carries. I guess I had to notify them like a week in advance. I thought I could’ve told them like in the middle of the week or something, but it didn’t work like that, I have to notify a week in advance, but I was never told that.” (Participant 18)*

Participants therefore described how access to OAT carries was crucial for OAT adherence and desistance from substance use. As such, many participants suggested being able to access and obtain OAT carries during their community transition period would have been advantageous:

*“I think [with] Suboxone, you shouldn’t have to wait so long for carries. That was my big thing.” (Participant 06)*

## Discussion

This study examined OAT transitions and related experiences among a sample of individuals with OUD who had been recently released from federal incarceration in Ontario, Canada. Examining OAT transition experiences among this vulnerable population provides unique insights for the Canadian correctional system context, and is particularly salient in light of the elevated health and substance use risks experienced by individuals during the period immediately following release from incarceration.

Participants described a variety of facilitators and barriers to continual engagement in community-based OAT post-release, as well as subsequent impacts on substance use. The study results highlight that the majority of participants remained engaged in OAT care upon community release; however, they reported several challenges in accessing and maintaining OAT care. Noteworthy facilitators of continued OAT engagement included the perceived psychological and physiological benefits of OAT, as well as the structure and routine of OAT regimens. These factors worked in tandem to reduce substance use cravings and reinforced continued OAT retention. Furthermore, trusted and supportive relationships with OAT providers were seen as key factors for reducing participants’ risk of relapse, and in turn, potential re-incarceration. Such positive client-centred supportive care delivered by non-judgemental OAT staff has been associated with increased treatment retention and success in other studies (Andraka-Christou et al., 2020; Fox, Jakubowski et al., 2019; Jackson, 2021; Maina et al., 2019; Marchand et al., 2018). For instance, one US study that examined the effectiveness of a transition clinic-based primary care program for justice-involved women who were re-entering into the community identified the importance of trauma-informed care and supportive staff in treatment engagement and retention (Thomas et al., 2019). Similarly, a qualitative study with individuals released from incarceration in Ontario found that relationships with health care providers played a pivotal role in encouraging continued health care access and engagement, and that this patient-centred support ultimately facilitated treatment and medication maintenance (Hu et al., 2020). As such, the establishment of positive and supportive patient-provider relationships appear to be an integral component for OAT adherence among correctional populations.

While participants described specific facilitators that contributed to OAT adherence, they also reported numerous barriers to accessing and maintaining OAT regimens once in the community. System-level issues included correctional release planning challenges, miscommunications between correctional facilities and OAT clinics, administrative issues, and difficulties securing financial OAT coverage which resulted in fragmented transitions and interruptions in OAT care for many participants. These challenges rendered participants vulnerable to relapse, release revocations, and potential re-incarceration—as evidenced by the high percentage of those who returned positive urinalysis drug tests during their supervision period and consequently had their release suspended and/or returned to custody. Many of these challenges have been well documented in previous research that has highlighted critical OAT interruptions upon community release, resulting in negative health and social consequences (Jamin et al., 2021; Joudrey et al., 2019; Vail et al., 2021). For instance, a recent qualitative study conducted in multiple European countries found that individuals released into the community encountered similar barriers to OAT engagement, particularly in the first few days post-release. Issues included challenges in release planning, problems accessing OAT clinics and obtaining prescriptions, in addition to administrative burdens such as complications with paperwork and securing financial OAT coverage, which resulted in relapse and an inability to receive required treatment (Jamin et al., 2021). A randomized control trial conducted in New York which examined the effectiveness of opioid pharmacotherapy formulations found that participants had difficulties balancing OAT and other reintegration requirements and noted the detrimental impact of scheduling conflicts (work, appointments, etc.), as well as financial constraints, such as the inability to pay for transportation to appointments; these issues resulted in early treatment dropout (Velasquez et al., 2019). Other studies have similarly identified logistical challenges to OAT access and maintenance such as limited transportation and timing conflicts between OAT appointments and other life and community release obligations (Owens et al., 2018). In the Ontario context specifically, insufficient correctional release planning, administrative issues related to enrolment in government social/financial assistance programs, as well as the lack of accessibility of OAT medications have been acknowledged as barriers that contributed to challenges accessing ongoing treatment, housing, and employment (Hu et al., 2020). Furthermore, a recent examination of federal community correctional centres in Canada identified many of the same barriers to successful community reintegration among individuals released from federal corrections, including a lack of adequate pre-release services and supports. The report specifically documented how individuals often arrive in the community without a provincial health card and a minimal (if any) supply of medication, after which they must access a clinic/family physician to refill their prescriptions. Yet, it takes a month or more to obtain identification, which leaves them in a vulnerable position without access to necessary medications (Office of the Correctional Investigator, 2014). Still, other studies have described interpersonal and social factors—including returning to drug use and criminally-involved social networks, environments, and lifestyles—as noteworthy risk factors for relapse, and these factors are particularly prevalent within the social ecologies surrounding OAT clinics (Binswanger et al., 2012; Bunting et al., 2018; Jamin et al., 2021; Larney et al., 2017; Velasquez et al., 2019).

### Policy implications

The results from this study underscore a number of key and critical implications for required policy amendments. As the most prominent barrier reported by participants, the fragmented community transition period—which unfortunately resulted in some participants experiencing high-risk OAT disruptions—requires necessary improvements to pre-release correctional discharge planning. Suggestions include the need to strengthen the coordination of care (e.g., referrals, information exchange, etc.) across different organizational sectors and establish direct linkages and partnerships between correctional institutions and key community organizations/service providers (e.g., OAT and addiction clinics; Binswanger et al., 2011; Eisenstein et al., 2020; Grella et al., 2020, 2021; Joudrey et al., 2019; Kouyoumdjian & Orkin, 2020; Office of the Correctional Investigator, 2014; Yatsco et al., 2020). Specifically, calls have been made to ensure that a rigorous process is in place to smooth the transition of individuals into the community, such as pre-release planning strategies and processes that ensure official documents (health cards, necessary medications and extended prescriptions) are available and provided pre-release where possible (Office of the Correctional Investigator, 2014).

Currently, it is up to correctional physician discretion whether to provide extended medical prescriptions to individuals pre-release, yet not all physicians are trained in addiction medicine and many do not feel comfortable providing prescriptions to individuals they are not able to monitor (Kouyoumdjian, Patel et al., 2018). For instance, a study examining OAT prescribing practices among provincial correctional physicians in Ontario identified a number of concerns that dissuaded them from prescribing OAT upon release, including sub-optimal linkages to community-based OAT providers, as well as an inability to ensure an appropriate clinical follow-up with the individual (Kouyoumdjian, Patel et al., 2018). These issues underscore the need for a systematic policy to ensure physicians are appropriately trained and feel comfortable and supported in providing OAT care. At a minimum, individuals whould be provided with access to pre-release prescriptions that can be used in the community until they can establish a new physician/OAT provider.

Additionally, since applications for government social assistance plans (e.g., welfare, disability, low-income prescription support) require extensive time periods for approval, and Ontario health authority policies currently restrict individuals from qualifying for some of these programs while they reside at community-based residential facilities (Ontario Ministry of Children CaSSM, 2021), efforts to initiate application processes for financial coverage should also occur pre-release where possible. Additionally, policies should be amended so that individuals have access to a range of financial options for medications/prescriptions during community supervision periods (Office of the Correctional Investigator, 2014).

Other recommendations include the uptake of post-release interventions such as robust case management though experienced system professionals (including those with lived correctional system experience, or “peers”), and the utilization of community transition clinics (i.e., primary and addiction care-points that engage patients in care coordination through direct referral from correctional institutions upon community release; Eisenstein et al., 2020; Howell et al., 2021; Hu et al., 2020; Jamin et al., 2021; Kendall et al., 2018; Kouyoumdjian & Orkin, 2020; Shavit et al., 2017; Wachino & Artiga, 2019). The use of progressive initiatives such as peer-support/case management and community transition programs have been associated with a number of benefits. These include improved health outcomes and decreased risk for substance use and criminogenic behaviours, as well as greater linkages to health care and treatment among individuals post-release (Banta-Green et al., 2019; Bellamy et al., 2019; Howell et al., 2021; Myers et al., 2018; Ray et al., 2021; Shavit et al., 2017; Waddell et al., 2020; Watson et al., 2017). Insights from the implementation of these programs with respect to their feasibility and applicability in Canadian contexts should be considered towards the improvement of post-release care for correctional populations with OUD.

Furthermore, study participants identified administrative policies at some halfway houses that prohibit possession of OAT medication carries onsite, which poses a major barrier to continued OAT adherence during community reintegration periods. These policies leave many individuals with no option but to make frequent (e.g., daily) visits to obtain their medication, thus increasing the likelihood of withdrawal and relapse if these appointments are missed, as well as via unnecessary exposure to substance use “hot spots”. To address this issue, organizational policies could be amended to allow halfway houses to store residents’ OAT medications onsite, as this is the status quo for other essential medications. Other recommendations for improving the system of care include the relaxation of OAT prescription guidelines to allow for unwitnessed OAT doses (i.e., via the provision of take-home carries) and the expansion of remote care delivery models (i.e., using telehealth and/or mobile options) for eligible patients. These can be combined with novel home-induction technologies that allow for patient-administered OAT dosing and adjunct online/telehealth monitoring (Gordon et al., 2019; MedicaSafe, 2019). Telehealth and mobile OAT delivery are low-barrier novel interventions that have shown promise for improving treatment access and retention, and can reduce barriers to treatment entry among remote and marginalized—including correctional—populations (Hall et al., 2014; Krawczyk et al., 2019; Krsak et al., 2020; O’Gurek et al., 2021; Stewart et al., 2021).

Finally, given the importance of patients’ personal agency and the ability to choose their desired OAT formulation and dosage as noted by study participants, a broader range of pharmacotherapy options should be offered to Canadian correctional populations. In Canada, buprenorphine-naloxone-based OAT has been considered the first-line treatment over methadone for OUD for a number of years (Bruneau et al., 2018; The British Columbia Centre on Substance Use (BCCSU), 2017). Alternative OAT formulations also exist such as slow-release oral morphine, and injectable diacetylmorphine or hydromorphone (Bruneau et al., 2018), yet these are only typically utilized in extenuating circumstances. As of 2019, injectable extended-release buprenorphine and a subdermal buprenorphine implant have also been approved for the clinical management of OUD (Health Canada, 2019). However, these formulations are currently underutilized, and were not yet being offered to study participants during data collection. The feasibility and outcomes of novel/alternative pharmacotherapies to treat OUD have been increasingly researched, particularly in the U.S, where a number of studies have examined the effectiveness of extended-release preparations (e.g., buprenorphine, naltrexone [an opioid antagonist], etc.) either alone or in comparison to other pharmacotherapies and found comparable post-release treatment retention and opioid use outcomes (Gordon et al., 2017, 2019; Vorspan et al., 2019; Waddell et al., 2021). While buprenorphine has been well-established, naltrexone has more recently been found to improve treatment retention, reduce opioid use (Bahji et al., 2020) and re-incarceration rates (Korownyk et al., 2019) among US-based correctional populations; however, naltrexone has not been approved in Canada for the clinical management of opioid use disorder and is currently only available for research purposes (The British Columbia Centre on Substance Use (BCCSU), 2017). Results from trials to-date emphasize that extended-release OAT formulations are a flexible option that may be beneficial for patients who are unable to attend frequent or daily in-person OAT appointments due to proximity concerns or employment and other life commitments (Chappuy et al., 2021; Compton & Volkow, 2021; Hard, 2021). In particular, these formulations may provide potential advantages for correctional populations who experience challenges balancing work and other community reintegration requirements. Additionally, community-based studies and randomized control trials have found favourable attitudes towards use of extended-release OAT among patients (Ahamad et al., 2015; Compton & Volkow, 2021; Lintzeris et al., 2021). These realities highlight the need for the expansion of OAT options in Canada, with the goals of reducing opioid use and related health and social risks, including re-incarceration, among correctional populations with OUD following community release.

### Limitations

This study involves a number of limitations. First, the study results are not generalizable beyond the small convenience-recruited study sample. Rather, the results highlight key themes described by a subset of recently released individuals on OAT in Ontario, Canada. Second, inherent biases in self-report data, such as recall, response, and negativity biases may exist. Whether an individual remains in OAT care or experiences substance use relapse post-release is not easily determined or verified; CSC monitors substance use relapse via community urinalysis and through community parole officers, but this information is not always known/available, and may therefore be underreported. Once someone has completed their supervision period, correctional institutions are no longer responsible for monitoring their outcomes in the community. As such, based on available data and the study design and scope, the results described represent only a temporal “snapshot” rather than a comprehensive account of participants’ community release experiences, and we were not able to capture any adverse outcomes such as overdose or mortality. Furthermore, due to the limited sample size of women, it was not possible to disaggregate the data by gender, and future research should focus on examining gender differences in regards to post-release OAT experiences.

## Conclusions

Canadian correctional populations experience a disproportionate burden of health challenges, including substance use dependence and specifically OUD. OAT is an increasingly utilized and essential intervention to reduce adverse health (e.g., morbidity and mortality) and social (e.g., recidivism) outcomes among incarcerated individuals with OUD; however, the post-release transitional period and structural aspects of the correctional system pose distinct challenges and barriers for continuous OAT engagement upon release. The results of this study emphasize a number of potential policy recommendations pertinent to OAT-related transitions that should be urgently implemented to improve health and reintegration outcomes among this high-risk population.

## Supplementary Material

Supplemental Material

## Data Availability

The datasets generated and/or analyzed during the current study are not publicly available due to the inclusion of personal identifying information, for which participants did not provide consent to share.

## Note

1. Participant identification numbers correspond to initial numerical codes provided at baseline assessment
