# Supplementary material for: Barriers and facilitators to opioid agonist treatment (OAT) engagement among individuals released from federal incarceration into the community in Ontario, Canada
Source: Int J Qual Stud Health Well-being. 2022 Jul 5;17(1):2094111. doi: 10.1080/17482631.2022.2094111 (PMC9258049; doi:10.1080/17482631.2022.2094111)
Supplement: Supplemental Material [file ZQHW_A_2094111_SM7742.docx]

# Appendix A: Follow-up qualitative interview guide

1. Please **DESCRIBE** your **GENERAL** life situation and experiences (e.g., related to health, relationships, work/finances, housing) **SINCE** your **RELEASE**:

**Prompts:**

- - How was the transition back into the community immediately after your release? Was the experience generally positive or negative?
- What has been going well for you?
- What has not been going well for you?

1. Please **DESCRIBE** your experiences with **OPIOID/** **OTHER DRUG USE** **SINCE** your **RELEASE**:

**Prompts:**

- Have you been using opioids/other drugs? Why/why not?
- **If participant currently using opioids/other drugs:** How has opioid/other drug use impacted the following areas of your life: Health? Relationships? Work/Finances? Housing?
- **If participant is not currently using opioids/other drugs:** What, if anything, has helped you manage your opioid/other drug use?

1. Please **DESCRIBE** your experiences with **OST SINCE** your **RELEASE**:

**Prompts:**

- Did you access OST after your release? Why/why not? Tell me about this experience.
- **If ‘accessed’ OST after release:** Have you remained engaged in OST since you accessed it? Why/why not? Tell me about this experience.
- Are you currently engaged in OST? Why/why not? Tell me about this.
- **If ‘currently engaged’ in OST:** How has OST been working for you? Is there anything you would change?
- Aside from OST, have you sought out or used any opioid or other drug use-related services since your release? If so, tell me about this.

1. Please **DESCRIBE** any **CHALLENGES** or **BARRIERS** that you have experienced regarding **OST**.

**Prompts:**

- Are there any specific factors that have made it difficult to access and/or remain engaged in OST? Please tell me about these.

1. Please **DESCRIBE** any **FACTORS, SUPPORTS** or **SERVICES** that have been important or helpful for you regarding **OST**.

**Prompts:**

- Are there any factors, supports or services that could have been helpful for you, or made your overall transition into the community easier, particularly in regards to OST?
- Are there any factors, supports or services that could have been helpful for you, or made your transition into the community easier in general?

1. Please **DESCRIBE** where you see yourself in a **YEAR**:

**Prompts:**

- Specifically regarding your opioid/other drug use?
- Specifically regarding OST?
- What do you want to see happen in your life over the next year, more generally?
